# Towards a Multidisciplinary Approach of ECG Screening in Children and Adolescents: A Scoping Review (2005–2025)

**DOI:** 10.3390/children12111468

**Published:** 2025-10-30

**Authors:** Giovanna Zimatore, Maria Chiara Gallotta, Matteo Campanella, Stavros Hatzopoulos, Piotr Henryk Skarzynski, Marta Ricci, Leonarda Galiuto

**Affiliations:** 1 Department of Theoretical and Applied Sciences (DiSTA), eCampus University, 22060 Novedrate, Italy; matteo.campanella@uniecampus.it; 2 Department of Life Sciences, Health, and Health Professions, Link Campus University, 00165 Rome, Italy; 3 Department of Physiology and Pharmacology “Vittorio Erspamer”, Sapienza University of Rome, 00185 Rome, Italy; mariachiara.gallotta@uniroma1.it; 4 Clinic of Audiology & ENT, University of Ferrara, 44122 Ferrara, Italy; sdh1@unife.it; 5 Heart Failure and Cardiac Rehabilitation Department, Faculty of Medicine and Dentistry, Medical University of Warsaw, 03-242 Warsaw, Poland; p.skarzynski@inz.waw.pl; 6 Institute of Sensory Organs, 05-830 Kajetany, Poland; 7 Department of Teleaudiology and Screening, World Hearing Center, Institute of Physiology and Pathology of Hearing, 05-830 Kajetany, Poland; 8 Department of Clinical and Molecular Medicine, Sapienza University of Rome, 00185 Rome, Italyleonarda.galiuto@uniroma1.it (L.G.); 9 Department of Cardiology and Angiology, Policlinico Umberto I University Hospital, 00161 Roma, Italy

**Keywords:** children, adolescents, pediatric, screening sports, cardiovascular system, heart rate variability, complex systems, sudden cardiac death

## Abstract

Background: The reported data on ECG screening are focused on the last two decades. The objectives of this review were bifold: (i) to identify, within a timespan of twenty years, the most recent literature data on cardiac screening in children and adolescents and (ii) to provide data on the procedures used. Methods: Queries were conducted using PubMed, Scopus, and Google Scholar databases for the time window of 2005–2025. The mesh terms used were “ECG”, “Universal Screening”, “Cardiac Pathologies”, “Heart Rate”, and “Sports Pre-participation Evaluation”. Only research articles and review papers were included. The standard English language filter was used. Successively, only research articles were selected. Results: Data from 14 papers were considered, reflecting the lack of information about subjects <16 years of age. Conclusions: The information on objective ECG screening measures is quite scarce, and it is an urgent need to introduce a multidisciplinary approach to differentiate between ECG physiological changes due to growth and ECG pathological changes due to early pathology.

## 1. Introduction

Cardiovascular health in childhood represents a cornerstone of lifelong health [1]; it requires a careful assessment, particularly during early school age and at the beginning of athletic participation, to establish a basis for safe physical activity [2,3]. In addition to collecting personal and family medical history and assessing signs and symptoms of cardiovascular disease (CVD), resting electrocardiography (ECG) is the key diagnostic tool in this setting [4].

Over the past few decades, interest in cardiovascular prevention during childhood and adolescence has grown significantly, driven by increasing awareness of the role that subclinical cardiac conditions may play in the development of adverse events, including sudden cardiac death in young individuals. In this context, ECG has received growing scientific attention and debate, both for its potential to detect early electrical or structural abnormalities and for the organizational, ethical, and economic implications of its widespread implementation.

Among the most studied ECG features are those indicative of cardiac fatigue (i.e., QTc prolongation, QT variability, -QTVI, and T-wave alternans), which are associated with autonomic dysfunction, electrical instability, and arrhythmic risk, particularly under physical or metabolic stress [2]. Data in the literature suggest that ECG, when integrated with tools such as echocardiography or cardiopulmonary exercise testing, may offer a more sensitive and dynamic assessment of cardiac function during the developmental stages.

Currently, there is no universally accepted diagnostic standard for the early identification of cardiac fatigue in the pediatric and adolescent population, nor any shared set of guidelines on the systematic implementation of ECG screening in these age groups. This has resulted in a substantial strategy heterogeneity across countries, with a wide variation in screening protocols, diagnostic technologies, and clinical criteria [2].

This scoping review aims to map the current cardiovascular screening practices in children and adolescents and contribute to the development of more effective, evidence-based preventive strategies. However, a single resting ECG recording may not provide a complete picture of cardiovascular health in children. Physiological growth can cause significant changes in the ECG waves and the corresponding intervals, and early signs of cardiac disease may appear gradually over time [4]. Therefore, longitudinal assessment of ECG changes during early childhood offers a more accurate evaluation of cardiac health. However, the criteria for normal pediatric ECGs are still under investigation. Similarly, it is unclear whether ECG changes over time indicate normal growth or early pathology [5].

In this scoping review, we collected and summarized existing literature published between 2005 and 2025, focusing on five key questions vital for guiding cardiovascular screening policies and practices in children and adolescents:

1. Which countries perform (universal and mandatory) ECG screening in the pediatric population;
2. In which settings is the ECG screening performed (school, pediatrician, sports medicine doctors, sports academies);
3. How many subjects are involved;
4. How many of them present an identifiable (by ECG) heart disease;
5. What are the most common protocols and ECG technologies used to assess early pathological signs.

This review is novel in its 20-year time frame and cross-national comparative scope, offering a comprehensive perspective on how screening approaches have evolved across different countries and contexts.

## 2. Materials and Methods

We conducted a comprehensive literature search using PubMed, Scopus, and Google Scholar, using a 20-year search window, covering the period from 2005 to 2025.

The literature search, conducted in January 2025, adhered to PRISMA 2020 guidelines (accessed January 2025, see Supplementary Materials) and used the following keywords (MeSH terms): “ECG,” “Universal Screening,” “Cardiac Pathologies,” “Heart Rate,” and “Sports Pre-participation Evaluation” in children (6–12 years) and adolescents (13–18 years). Only research articles and reviews were considered suitable. An English language filter was applied. The primary criterion for material quality was publication in a peer-reviewed journal, but other criteria were also considered. Additionally, strict adherence to established screening protocols and clearly outlined methodologies was considered (e.g., all newborns had to be screened for hearing abnormalities). Table 1 presents the selection criteria.

Bibliographic data were collected from PubMed, Scopus, and Google Scholar. Two independent reviewers (G.Z.) and (M.C.) went over the available material (396 papers) successively; only research articles were selected, and the final number of eligible papers was distilled to 14.

**Figure 1:**
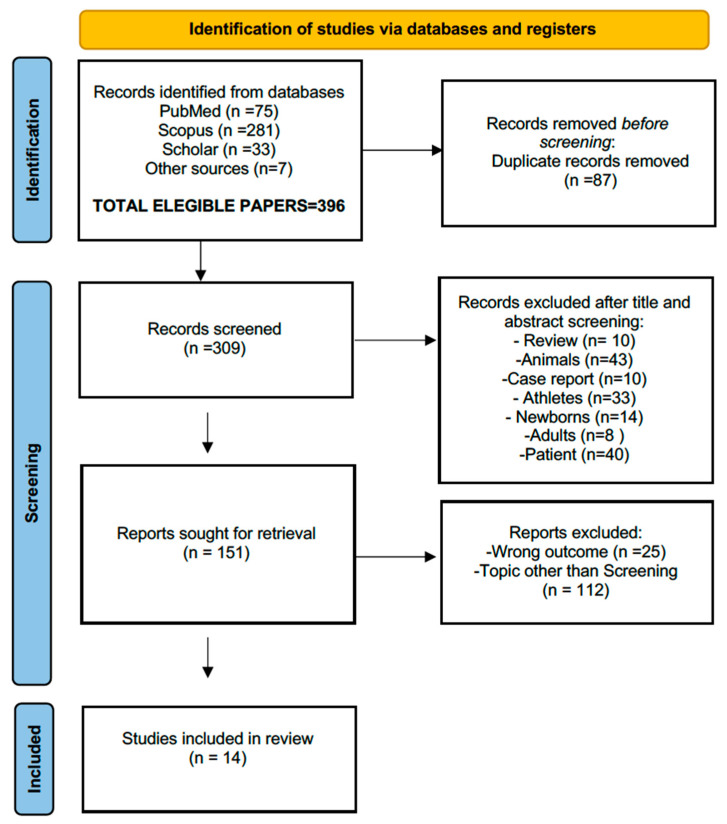
Flow diagram of the literature search according to the PRISMA criteria (http://www.prisma-statement.org/, accessed on 25 January 2025), illustrating the steps followed in the overall manuscript-selection process. After applying the selection criteria, the initial 396 manuscripts were reduced to 14.

The PRISMA flowchart process is reported in Figure 1; after the initial screening of the manuscript’s title and abstract, in the second phase, we considered the clarity of the study objectives, the transparency of the screening protocols, the sample size, the population representativeness, and finally, the completeness of the reported outcomes. Studies were also appraised for (i) the methodological consistency with established screening guidelines (i.e., by the American Heart Association -AHA-, or the European Society of Cardiology -ESC-, see Appendix A for additional information); and (ii) the relevance of their findings related to the research questions. This approach ensured that only studies providing sufficient methodological detail and reliable outcome data were included in the pool of suitable manuscripts. The selected studies for review are reported in the next paragraph.

## 3. Results

**Figure 2:**
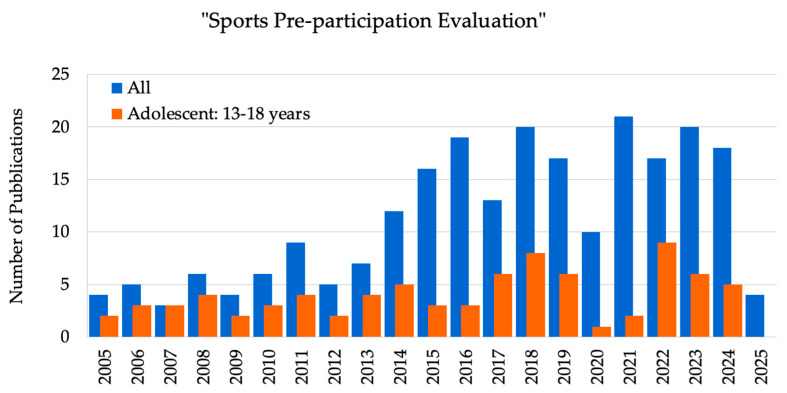
Annual number of publications on ‘Sports Pre-participation Evaluation’ between 2005 and 2025. The blue line represents the total number of publications across all age groups, while the orange line specifically indicates publications focusing on adolescents (13–18 years).

A total of 396 articles were assessed based on the search formula and screening procedure described above. Since 2012, research on ECG-based screening and sports pre-participation evaluation has been increasing annually (Figure 2). The number of yearly publications rose to 20 papers in 2018 and then remained stable, except during the COVID-19 pandemic period, demonstrating the relevance and potential of ECG-based screening. However, there is a lack of information about children (6–12 years) and adolescents (13–18 years) during the COVID-19 pandemic period (2020–2021).

The data were organized alphabetically by country of origin, and the results are summarized in Table 3 and Table 4 at the end of this section.

Figure 2 depicts the publication trends related explicitly to sports pre-participation evaluation in both adult and adolescent groups. The emphasis on adolescents is justified for several reasons. First, adolescents are in a critical stage of physiological development, undergoing hormonal, cardiovascular, and musculoskeletal changes that may increase their risk. Additionally, they often engage in high-intensity sports, making cardiovascular health monitoring essential to prevent injuries and support healthy growth. Moreover, an increasing global awareness of sudden cardiac death (SCD) among young athletes has brought greater attention to the legal and ethical responsibilities of sports organizations [4,5,6] (see also Appendix A).

The scientific interest and the volume of data on adolescents have recently increased, as shown by the growing number of publications. Five of the fourteen selected papers of this review (36%) were published in the last five years; the studies included both children (6–12 years) and adolescents (13–18 years).

Of the 648,172 subjects included, 572,563 (88%) were 6–7 years old. Two of the fourteen selected papers (14%) covered age in the range from 6 to 10 years, and two from 6 to 12 years. Only one paper was based on subjects from 0 to 18 years (14,530 subjects). Finally, three papers (21%) covered 11,702 (2%) subjects from 12 to 18 years.

Most studies focused predominantly on school-entry-age populations, reflecting the strategic importance of this developmental milestone for implementing systematic cardiovascular screening programs.

All studies reported the use of ECG for detecting heart disease. Among them, 2 studies (14%) used both echocardiography and ECG.

In six studies (43%), AHA guidelines were used; in two studies (14%), both AHA and the pre-participation cardiovascular evaluation (PPCE) were implemented. Finally, of the 14 studies, seven (50%) were conducted in the EU, two (14%) in the USA, and one in China, Japan, Taiwan, Indonesia, and the Pacific Islands.

The multi-step screening approach, shown in Table 2, aims to optimize early detection of congenital and acquired heart diseases in the school-aged population, facilitating timely referral and management. Table 2 depicts the main measures used and the 14 papers for this review are reported in Table 3 and Table 4.

### 3.1. China

In China, cardiac screening is not widely used, especially in remote areas with limited medical resources, due to issues related to cost, distance, and human resources.

The goal of Liu et al. [7] was to develop a cloud-based system that could be used to screen students for heart disease in large numbers in rural areas with limited access to healthcare. The study included data from 5842 schoolchildren aged 6–18 years from elementary and high schools in Taiwan, who participated in mandatory cardiac screening programs. The protocols consisted of combined cardiac auscultation, performed by trained nurses, and 12-lead ECG with automated interpretation. The latter was integrated into a crowdsourcing telemedicine platform connecting school health centers with cardiologists for real-time consultation and abnormality verification. The hybrid screening model achieved a 98.7% completion rate with 2.1% abnormal findings requiring cardiology referral, demonstrating effective large-scale pediatric cardiac screening through technology-enhanced healthcare delivery systems.

### 3.2. Germany

Duman et al. [8] assessed data from 11,487 German children aged 7 to 18 years who were referred to the clinic for pre-participation sports screening between September 2017 and December 2021. All children underwent a cardiovascular evaluation, including assessment of personal and family medical history. They were evaluated with echocardiography and ECG. Initially, 34 out of 11,487 (0.29%) subjects were restricted from participating in sports. Cardiac arrhythmias were found in 23 of these 34 patients. Three subjects showed ventricular extrasystoles, one had ventricular tachycardia, and fifteen were diagnosed with Wolff–Parkinson–White (WPW) syndrome. Notably, one of these patients also experienced ventricular tachycardia. Additionally, one subject had ST elevation and a pre-diagnosis of coronary artery disease, while four other subjects exhibited long QT syndrome.

Positive echocardiography and ECG results show a relatively low ratio. However, ECG screening alone was able to identify asymptomatic individuals (0.05%) who might be at risk for sudden cardiac death.

### 3.3. Greece

In a prospective study, Bagkaki et al. [9] explained that early detection and prompt preventive measures were made possible by screening children for CVD and its associated risk factors. In their study, 944 voluntary third-grade primary schoolchildren from 150 schools in the Crete region of Greece were evaluated over six years (2018–2024). The study included three phases: (i) the primary phase involved personal and family questionnaires and physical evaluations; (ii) the second phase involved data verification; and (iii) the third phase was related to tertiary center evaluation, where the subjects were assessed with echocardiography and repeated 12-lead ECGs. A final diagnostic assessment revealed that 69 (7.3%) of the participants had abnormal CVD findings. These include minor or trivial structural heart disease in 23 subjects (2.4%) and abnormalities identified due to abnormal cardiac auscultation in 17 subjects (1.8%). ECG abnormalities were reported in 29 subjects (3%); six of those (0.6%) were potentially significant, including one case of genetically confirmed channelopathy (LQT syndrome) caused by a novel KCNH2 pathogenic mutation.

### 3.4. Indonesia

Dinarti et al. [10], in a study involving 6116 Indonesian elementary school students, reported that the use of the 12-lead ECG and cardiac auscultation was a viable method for primary screening. The study identified 5.38% of elementary school students who may have had congenital heart disease. During a secondary screening, 6.9% (18 out of 260) of the heart abnormalities found were confirmed by transthoracic echocardiography, including congenital septal defects (2.7%) and valve abnormalities (4.2%).

The overall reported prevalence of heart abnormalities was 0.29% (18 out of 6116).

### 3.5. Italy

In Italy [11], a nationwide program of pre-participation screening was started in 1982 for everyone starting competitive sports. Athletes’ personal and family histories, physical examinations, and a 12-lead electrocardiogram (ECG) are all part of the screening protocol (see Table 2 A–F).

Only subjects who show positive results from the initial evaluation are asked to undergo further testing, such as echocardiography or exercise testing. Additionally, to provide sufficient sensitivity and specificity for identifying athletes with potentially dangerous cardiomyopathy or arrhythmia at risk of athletic-field death, this screening algorithm has been used for the pre-participation evaluation of millions of Italian athletes for over 25 years. It has also significantly reduced the mortality rate among young competitive athletes (by about 90%), mainly by preventing sudden death from cardiomyopathy. Furthermore, since 2013, all individuals involved in recreational physical activities should have an ECG recorded and analyzed. Telecardiology has proven to be a useful and cost-effective tool for ECG screening [12,13].

### 3.6. Japan

In Japan, nationwide school-based ECG screening for CVDs has been implemented since 1995, following its formal enactment into legislation [14]. Yoshinaga et al. [15] reported on the screening program conducted between 2008 and 2013 in Kagoshima, Japan, involving a total of 33,051 first graders and 34,751 seventh graders, aged 6 and 12 years, respectively. The screening process consisted of three examination steps: an initial screening followed by second and third exams, focusing on determining the probability of diagnosing the Long-QT (LQT) syndrome in children and adolescents.

In 2013, the Heart Rhythm Society (HRS), the European Heart Rhythm Association (EHRA), and the Asia Pacific Heart Rhythm Society (APHRS) (HRS/EHRA/APHRS) published a Consensus Statement on the diagnosis and management of patients with inherited primary arrhythmia syndromes. The HRS/EHRA/APHRS criteria are considered to evaluate the probability of diagnosing LQTS.

### 3.7. Pacific Islands

The study of Chatard et al. [16] analyzed the results of the PPCE on athletes from the Pacific Islands. Between 2012 and 2015, 2281 athletes from 14 Pacific Islands underwent PPCE, which included family and personal history, a physical examination, and a 12-lead resting ECG. Additionally, all athletes completed a questionnaire including the main items from the AHA 12-element recommendations, while the physical examination was based on the Consensus Statement from the European Society of Cardiology sports group. The ECG analysis was initially carried out using the Seattle criteria. Fifteen percent of the screened individuals required further investigations, revealing cardiovascular abnormalities in 3.9% of the cases, with 0.8% found to have conditions associated with a risk of SCD. A total of 14.6% of cases showed clinical abnormalities during the physical examination or from their medical history. ECG abnormalities varied according to ethnicity, age, and training load. Overall, 3.9% of the athletes were diagnosed with a cardiovascular condition, while 0.8% had a condition associated with an increased risk of SCD, such as hypertrophic cardiomyopathy, Wolff–Parkinson–White syndrome, long QT syndrome, and severe valvular diseases. Despite the screening, three cases of SCD occurred during the five-year follow-up period. All athletes presenting abnormal findings were offered further complementary cardiac screening; however, in some low-income Pacific Islands, some of these additional investigations, although highly recommended, could not always be performed, either due to economic reasons or the lack of sports cardiologists or specialized medical facilities.

### 3.8. Spain

The study by Greciano et al. [17] involved 641 children from the Balearic Islands. This was an observational, descriptive, multicenter, cross-sectional study conducted from April 2021 to January 2022. The paper reports all the record forms used for medical assessments, physical examinations, and 12-lead ECGs, including twelve characteristics (normal or altered). The sensitivity of primary care physicians’ ECG interpretation for identifying an ECG indicative of sudden cardiac death risk was 29%, with a positive predictive value of 45%. The study lays the groundwork for future SCD risk screening in children carried out by primary care physicians. The authors hope that this screening will be integrated into routine healthy-child check-ups by primary care pediatricians and not limited only to children who participate in organized sports.

### 3.9. Switzerland

In Switzerland, screening is recommended starting at age 12, beginning with a medical history and physical examination. Before this age, an individual assessment of athletic performance is advised. From age 15 or upon reaching post-pubertal development, it is recommended to include a resting 12-lead ECG in PPCE [18].

Medical history and physical examination remain the foundation of PPCE and should be conducted using standardized tools, such as the AHA 14-element screening. Resting ECG improves sensitivity and negative predictive value for detecting SCD-related conditions; it is endorsed by the ESC and is widely used in Switzerland as part of the screening process. Echocardiography has not demonstrated additional diagnostic benefit in routine screening and is not recommended as a first-line tool. The international criteria for electrocardiographic interpretation in athletes are considered appropriate for use in the pediatric and adolescent population. ECQ-inclusive PPCE is more cost-effective than history and physical examination alone, especially considering the long-term benefits of early detection and intervention.

In the study of Perrin et al. [19], 287 children and adolescents were screened from various competitive sport disciplines, undergoing mandatory pre-participation cardiovascular screening at specialized sports medicine centers. Standardized 12-lead ECG was interpreted using the contemporary international screening criteria (ESC, European Society of Cardiology), with systematic classification of normal training-related adaptations versus pathological findings requiring further investigation. This procedure identified abnormal ECGs in 8.3% of athletes (significantly lower than older criteria), with 2.1% requiring additional cardiac evaluation, thus improving the specificity while maintaining an adequate sensitivity for detecting potentially life-threatening cardiovascular conditions.

### 3.10. Taiwan

Liu et al. [20] presented data from cardiac screening results for 566,447 school-age children in Taipei between 2003 and 2014. The cardiac screening included questionnaire surveys (Stage I, see C in Table 2), a simplified phonocardiography test, and a simplified ECG test; a pediatric cardiologist performed a physical examination and auscultation (in Stage II) on all children with abnormal results from Stage I screening; in Stage III, all referred children from the previous phase were sent to pediatric cardiologists for additional testing.

Analyses using decision trees and logistic regression were also conducted, and 685 children (0.12%) were identified as being at high risk of SCD. Unlike earlier findings, the study of Liu et al. [20] found that self-identified symptoms and family history of SCD did not influence the identification of high-risk cases with SCD. A simplified ECG test was shown to be the most significant variable for detecting high-risk SCD in logistic regression analysis (for more details, see Appendix B).

### 3.11. Turkey

Dag et al. [21] conducted a retrospective study to evaluate the effectiveness of sports pre-participation cardiovascular screening in Turkish children and adolescents under 18 years of age, following the 12-element screening guidelines of the AHA. The aim was to identify cardiac conditions that could lead to sudden cardiac death in asymptomatic young athletes. Between April 2016 and January 2018, they included 974 children referred to a pediatric cardiology outpatient clinic for a medical fitness certification before starting sports activities. Data were collected from fully completed case report forms, which included personal and family medical histories, physical examinations, anthropometric measurements, and results from both ECG and transthoracic echocardiography (TTE). The study emphasized the importance of a structured cardiovascular assessment prior to sports participation. Although the AHA guidelines recommend taking medical history and performing physical exams, these methods have relatively low sensitivity. In the study of Dag et al. [21], five potentially dangerous conditions would have gone unnoticed without ECG and TTE. Incorporating ECG into screening protocols significantly improves sensitivity. However, some concerns remain regarding the possible false positives, especially in athletes, due to physiological adaptations. Also, concerns were expressed for the false negatives of patients with serious cardiac issues who present normal ECG results.

The study supported the integration of ECG and, when clinically indicated, echocardiography into screening protocols to improve the diagnostic accuracy and reduce the risk of SCD in young athletes.

### 3.12. UK

Between 1996 and 2016, over 110,00 young football players aged 15 to 17 years, who were enrolled in the youth academies of professional clubs affiliated with the English Football Association, underwent mandatory cardiovascular screening [22]. The screening protocol included a health questionnaire, a physical examination, a 12-lead ECG, and TTE. Athletes were categorized into three groups: a group with normal results, a group with findings needing further evaluation, or a group presenting data of a cardiac disorder. Detected conditions were further classified as either linked to a risk of SCD or as other structural abnormalities.

When abnormalities were suspected, athletes were referred to specialized centers for further testing, including cardiac magnetic resonance imaging, maximal exercise testing, 24-h Holter monitoring, and electrophysiological studies, if indicated.

The program identified 42 athletes (0.38%) with cardiac conditions associated with a risk of sudden death. The most frequently diagnosed conditions included hypertrophic cardiomyopathy (5 cases), long QT syndrome (3 cases), coronary anomalies (2 cases), and Wolff–Parkinson–White syndrome (26 cases). An additional 225 athletes were found to have less severe cardiac abnormalities, such as congenital septal or valvular defects.

Athletes at risk for SCD were disqualified from competitive sports. All cases were monitored over time. During an average follow-up of more than 10 years, 23 deaths occurred, with 8 being cardiac-related and happening during physical activity. Notably, 6 of the 8 athletes who died showed no detectable abnormalities at initial screening, underscoring the limitations of cardiovascular testing during adolescence when some conditions may not yet be visible.

The total cost of the program was about USD 4.3 million. The average cost per diagnosis of a potentially deadly cardiac condition was roughly USD 103,000, while diagnosing any cardiac abnormality cost approximately USD 16,000.

### 3.13. USA

A national dataset with over 12.3 million subjects aged 5 to 21 years was analyzed to assess the effectiveness of pre-participation screening protocols from 2005 to 2010. The AHA guidelines were widely adopted, especially in school-based and pediatric outpatient settings. The screening process included family and personal history assessments and physical exams, with ECG added in certain cases. Despite the large scale of screening, differences in implementation across states were observed. Studies based on this dataset highlighted the limitations of non-ECG screening and called for broader use of ECG to enhance diagnostic sensitivity, especially in asymptomatic individuals [6].

In Minnesota, a large-scale retrospective study analyzed over 4.4 million adolescents aged 12–18 years from 1986 to 2011 [23]. Pre-participation cardiovascular screening followed the AHA 14-element guidelines, which included reviewing personal and family medical histories and performing a physical exam. Although the protocol did not always include an ECG, later analyses suggested that many potentially fatal cardiac conditions could have been missed with only history and physical exam [23,24]. The findings fueled ongoing debate about including the ECG in routine screening and played a crucial role in shaping national policies on cardiovascular risk assessment for youth athletes.

**Table 3:** The 14 eligible papers after the filtering process: The national data are listed alphabetically. Meaning of the used terms: CHD, congenital heart disease; CCFT, Cardiac Children’s Foundation Taiwan; YPPHE, Youth Physical and Public Health Evaluation; AHA, American Heart Association; ESC, European Society of Cardiology; PPCE, pre-participation cardiovascular evaluation; HRS, Heart Rhythm Society; EHRA, the European Heart Rhythm Association; and APHRS, the Asia Pacific Heart Rhythm Society (see Appendix B, Appendix C and Appendix D).

n	Country	Sample Size	Pre-Sport/ Young Athlete	Start Date	Project, Method	Age (Years)	Authors (First Name)	Pub. Year
1	China (Xinjiang, NW)	14,530	no	2018–2019	CHD, See Table 2 (A–G)	0–18	Liu F [7]	2023
2	Germany	11,487	yes	2017–2021	See Table 2 (A–F)	7–18	Dunan [8]	2024
3	Greece	944	no	2018–2024	CVD screening AHA guidelines	6–10	Bagkaki [9]	2025
4	Indonesia	6116	no	2018–2019	See Table 2 (A–G)	6–7	Dinarti [10]	2020
5	Italy	247	yes	2010–2014	YPPHE	13–18	Adami [11]	2008
6	Japan	33,051	no	2008–2013	screening program HRS/EHRA/APHRS criteria	6–12	Yoshinaga [15]	2016
7	Pacific Islands	2281	yes	2012–2015	PPCE, AHA guidelines	18–21	Chatard [16]	2019
8	Spain	640	yes	2021–2022	ESC sports group	6–12	Greciano [17]	2024
9	Switzerland	287	yes	2013–2016	PPCE, AHA guidelines	14–24	Perrin [19]	2016
10	Taipei/Taiwan	566,447	no	2003–2014	SCD screening CCFT program	6–7	Liu H [20]	2020
11	Turkey	974	yes	2016	AHA guidelines	8–14	Dag [21]	2019
12	UK	11,168	yes	1996–2016	See Table 2 (A–F)	12–18	Malhotra [22]	2018
13	USA (Minnesota)	4,440,161 (DS)	yes	1986–2011	screening program AHA guidelines	12–18	Maron [23]	2016
14	USA	12.3 million (DS)	no	2005–2010	Retrospective study AHA guidelines	5–21	Burns [24]	2015

DS: retrospective analysis of datasets; columns in light gray: children; in gray: adolescents; white: both children and adolescents.

**Table 4:** Summary of pediatric and adolescent cardiovascular screening studies from the 14 eligible papers.

n	Country	Authors (First Name)	Prevalence of Abnormal ECGs	Sensitivity/Specificity of Screening	Key Findings
1	China (Xinjiang, NW)	Liu F [7]	2.1% abnormal findings requiring cardiology referral; 16.5‰ overall prevalence of CHD	NA	Technology-enhanced telemedicine model is feasible for large-scale rural screening.
2	Germany	Dunan [8]	0.05% abnormal ECGs identified asymptomatic at-risk individuals; 0.29% were restricted from participating in sports	NA	Low prevalence, but ECG is useful for detecting asymptomatic at-risk youth.
3	Greece	Bagkaki [9]	7.3% abnormal CVD; 3% ECG abnormalities (29/944) (0.6% potentially significant)	NA	Early detection of minor and potentially significant CVD through school screening.
4	Indonesia	Dinarti [10]	0.29% overall prevalence of heart abnormalities (18/6116)	NA	Feasibility of ECG + auscultation as a primary screening tool in schools.
5	Italy	Adami [11]	4.5% cardiovascular abnormalities	High sensitivity and specificity demonstrated over time	Adolescent elite athletes, although capable of astonishing performances, may harbor clinical conditions that require constant follow-up and monitoring.
6	Japan	Yoshinaga [15]	Probability of diagnosing LQTS is 1:3300 in subjects aged 6 years and 1:1000 in those aged 12 years	NA	HRS/EHRA/APHRS criteria to evaluate LQTS.
7	Pacific Islands	Chatard [16]	3.9% cardiovascular abnormalities, 0.8% at risk of SCD; a high prevalence of RHD (1.5%)	NA	Ethnicity and training influenced ECG variability; limited follow-up resources.
8	Spain	Greciano [17]	92,64% normal ECG	SCD risk detection: Sensitivity: 29%; Positive Predictive Value: 45%	Primary care ECG interpretation showed moderate accuracy for SCD risk detection.
9	Switzerland	Perrin [19]	1.4% abnormal ECGs (revised Seattle criteria)	Improved specificity with maintained sensitivity using modern criteria	Modern ECG criteria reduce false positives while maintaining accuracy.
10	Taiwan	Liu H [20]	0.12% high-risk for SCD identified via simplified ECG	NA	Questionnaire survey and Simplified ECG identified key SCD predictors in large-scale school screening.
11	Turkey	Dag [21]	3.1% had a pathological finding according to the AHA screening guide (15.8% had sinus arrhythmia, 0.2% had ventricular extra beats, and 0.2% had LQT)	ECG improved sensitivity: combined ECG + TTE increased diagnostic accuracy	Adding ECG and TTE improved the detection of life-threatening conditions.
12	UK	Malhotra [22]	0.38% with cardiac conditions linked to SCD	NA	Mandatory screening detected at-risk athletes, but some conditions remained undetected.
13	USA	Maron [23]	Incidence of SCD events: 1/150,000 participants/ys	AHA-based screening showed low sensitivity without ECG	National data support adding ECG to improve the detection of asymptomatic cardiac risks. Based on autopsy data, only about 30% of the SCDs were due to diseases that could be reliably detected.
14	USA	Burns [24]	0.8% of cardiac disease	ECG inclusion improves sensitivity	With a high rate of detecting heart illness, these real-world data show that community providers selectively employ the ECG as part of the PPE.

Explanation of terms: NA, Not Available; RHD, Rheumatoid Heart Disease; CHD, congenital heart disease; SCD, Sudden Cardiac Death; CVD, Cardiovascular Disease; Note the significant geographical differences: the highest birth prevalence of CHD is in Asia (9.3‰), followed by Europe (8.2‰) and North America (6.9‰), and the lowest is in Africa (1.9‰) [25]; PPE, pre-participation history and examinations.

## 4. Discussion

Resting ECG remains a valuable tool for identifying congenital heart diseases and cardiac arrhythmias that might otherwise remain undiagnosed until the occurrence of adverse events. However, the widespread implementation of ECG screening in the pediatric population poses several logistical and practical challenges. This scoping review analyzed studies published over the past 20 years, focusing on cardiac screening in children and adolescents, with the aim of evaluating the effectiveness of ECG-based programs and informing future cardiovascular screening policies.

The first evidence indicates that, while ECG screening is not compulsory for the general pediatric population, it is mandated as part of pre-participation cardiovascular evaluation in young athletes. Regarding screening settings, most research efforts have been focused on school-age populations (first entry: 6–7 years) [10,20]. This emphasis reflects a strategic approach to early detection during a developmental stage when children transition from family-based to institutional healthcare. Schools represent effective and cost-efficient platforms for large-scale pediatric screening, aligning with established public health frameworks designed to maximize population reach and minimize disparities in access to preventive cardiovascular care.

In terms of screening modalities, current evidence remains insufficient to support the routine use of echocardiography for all children, despite its ability to detect a broader range of structural abnormalities. Instead, the integration of resting ECG as a primary screening tool, particularly when combined with medical history and physical examination, appears to offer an optimal balance between diagnostic yield, feasibility, and resource utilization. With respect to athletic populations, pre-participation screening is widely recommended to reduce the risk of SCD among young athletes [20,22,23]. Although SCD is rare in this group, its dramatic and unexpected nature underscores the importance of preventive strategies. The reviewed screening programs typically included a resting 12-lead ECG, physical examination (including blood pressure measurement), and assessment of family and personal medical history (see Table 2 A–F). Such comprehensive evaluations, when conducted by qualified professionals (pediatricians, family physicians, cardiologists, or sports medicine specialists), can identify at-risk individuals early and significantly contribute to reducing sport-related cardiac mortality (more details can be found in Appendix D).

**Figure 3:**
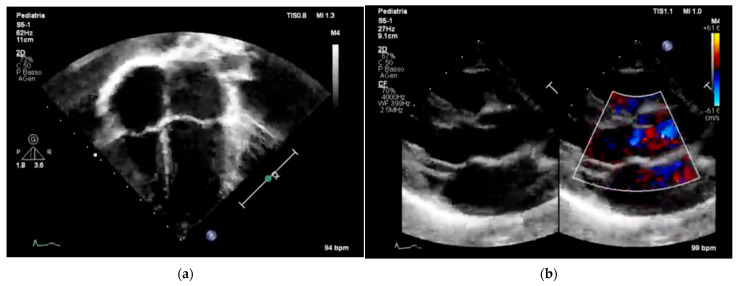
Two representative examples of echocardiography in pediatric subjects: (**a**) four-chamber pediatric view with apex down in 6-year-old children; (**b**) parasternal long axis view of a 6-year-old child; black and white on the left and color Doppler flow on the right.

**Figure 4:**
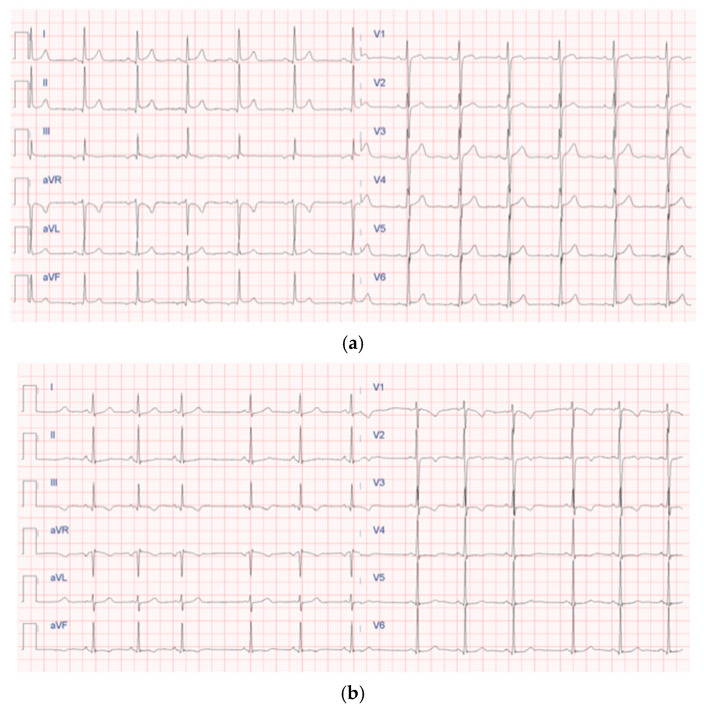
Two representative examples of electrocardiography in pediatric subjects: (**a**) normal pediatric 12-lead electrocardiogram; (**b**) sinus arrhythmia and negative T waves in leads V1–V3 as a normal finding in 3-year-old children.

Figure 3 and Figure 4 underscore the importance of age-specific interpretation in pediatric cardiology to distinguish normal developmental findings from true abnormalities.

These representative images highlight the importance of pediatric-specific expertise in interpreting both echocardiograms and electrocardiograms. In children, normal anatomical and electrophysiological features change with age, and misreading them can lead to overdiagnosis or unnecessary restrictions on physical activity. Additionally, including color Doppler imaging in echocardiography (Figure 3b) emphasizes the importance of evaluating hemodynamic function alongside structural integrity, especially in screening protocols designed for early detection of CHD [7] or functional issues. Similarly, the ECG patterns shown in Figure 4b display the typical pattern of physiological T-wave inversion in the right precordial leads (V1–V3), which is common in children under 8 years old. This underscores the need for age-specific ECG interpretation criteria to prevent normal variants from being mistaken for pathological conditions.

Overall, Figure 3 and Figure 4 emphasize the importance of combining imaging and electrophysiological tools in pediatric cardiovascular screening while also highlighting the crucial role of *age-specific norms* and clinical context in ensuring accurate, efficient, and cost-effective screening results.

Five key questions (in italics) outlined in the introduction were systematically addressed to provide a comprehensive synthesis of current evidence:

*Which countries perform (universal and mandatory) ECG screening in the pediatric population?* Japan [15] is the clearest and best-documented example of universal, population-wide mandatory ECG screening of all children (school-age or all neonates/children).

Across the studies reviewed, ECG screening practices for children and adolescents vary substantially between countries, reflecting differences in healthcare infrastructure, national policies, and available resources. Nations with structured and mandatory programs (e.g., Italy [11] and the UK) have demonstrated greater success in identifying at-risk individuals and preventing adverse outcomes, whereas low- and middle-income countries have shown that ECG screening remains feasible, though often constrained by economic and logistical barriers.

*How many subjects are involved? And in which settings is the ECG screening performed (school, pediatrician, sports medicine doctors, sports academies)?*

The size and composition of screened populations differ widely, from small cohorts [11] to millions of participants [24], depending on whether screening is mandatory, targeted, or voluntary. Screening settings also vary, including schools, pediatric clinics, and sports medicine centers, underscoring the importance of integrating screening into accessible community or educational contexts.

*How many of them present identifiable (by ECG) heart disease?*

Reported prevalence rates of cardiac abnormalities range from 0.29% to 3.9%, with serious conditions such as hypertrophic cardiomyopathy, long QT syndrome [15,21], and Wolff–Parkinson–White syndrome representing a small but clinically significant subset. These findings highlight the variable sensitivity and specificity of ECG interpretation in pediatric populations and emphasize the need for standardized diagnostic criteria and improved professional training, potentially supported by AI-assisted ECG interpretation.

*What are the most common protocols and ECG technologies used to assess early pathological signs?*

Finally, the screening protocols and technologies employed remain heterogeneous, with notable differences between European and U.S. approaches. While many programs follow elements of the ESC or AHA guidelines, there is no universally accepted protocol. The increasing use of digital 12-lead ECG systems and telemedicine platforms offers promising advances in scalability and accuracy. A globally harmonized, evidence-based framework adapted to local resources could improve consistency, cost-effectiveness, and equity in pediatric cardiovascular screening.

### 4.1. Limitations of the Study

This study has certain limitations, such as potential selection biases and the lack of long-term follow-up data to assess the true impact of ECG screening on children. Additionally, variations in how healthcare professionals interpret ECGs may affect the applicability of the results.

Furthermore, publications in local journals not indexed in PubMed or Scopus might not have been considered during the manuscript evaluation process. However, because of their lack of an impact factor, including such manuscripts could have compromised the quality criteria set for this scoping review.

### 4.2. Future Directions

Future analyses could aim to validate standardized assessment methods for PPCE in pediatric populations. The integration of machine learning algorithms for ECG analysis could further enhance screening accuracy. For example, convolutional neural networks (CNNs) have proven effective in classifying complex medical imaging data, such as chest X-rays for COVID-19, pneumonia, and normal conditions [26], or in detecting subtle patterns in multi-dataset forensics problems [27]. Similarly, advanced architectures like LSTM networks, optimized for real-time inference on hardware-constrained devices, demonstrate the potential of AI models for continuous physiological monitoring, such as UAV engine telemetry [28]; this approach could inspire real-time ECG monitoring and early anomaly detection in at-risk populations.

Furthermore, early predictors of CVDs, including molecular biomarkers and physiological indicators like HRV [2], may be valuable for early detection of conditions involving cardiovascular overload, such as those caused by intense physical activity, chronic stress, or underlying health issues. Although physical activity is not directly associated with increased cardiac mortality in young individuals, it can trigger sudden cardiac death in those with pre-existing cardiovascular problems, often of genetic origin, that make them susceptible to dangerous arrhythmias during exercise [29,30]. The higher rate of sudden cardiac death seen in athletes compared to non-athletes is mainly due to the hidden presence of inherited cardiomyopathies, which can remain asymptomatic until intense exertion occurs [31]. More research is needed to understand how rare genetic variants increase the risk of sudden death among adults, and targeted genetic screening should be employed to identify these risks [32,33].

The decline in mortality started after mandatory screening was introduced and persisted throughout the later screening phase. Therefore, a multidisciplinary assessment that includes a health questionnaire, lifestyle habits questionnaire, physical examination, physical fitness test, and ECG could be very helpful for initial screening to identify at-risk individuals, who can then undergo more advanced diagnostic procedures.

## 5. Conclusions

ECG screening in children shows promise for early detection of CVDs and may help reduce the risk of sudden cardiac death in young athletes. This scoping review highlights the growing international interest and various methods of ECG screening strategies in the pediatric population across different countries over the past twenty years.

Discrepancies in policies concerning the cardiovascular assessment of asymptomatic children were found. While some countries endorse universal screening strategies, others prefer a more targeted approach based on individual risk factors. Although there is consensus on the importance of early detection of CVD in young athletes, significant differences persist between countries in how screening programs are conducted and how resting ECGs are interpreted in children. Further research is needed to establish clear ECG interpretation standards with *age-specific norms* and to develop standardized screening protocols, aiming to create a consistent, evidence-based strategy that balances early detection with reducing unnecessary interventions.

Future advancements in technology, such as AI-driven ECG analysis, could improve diagnostic accuracy and decrease false positives, boosting the efficiency of screening programs. Furthermore, standardizing screening guidelines across various healthcare systems may help ensure fair access to cardiovascular risk assessment, making screening more accessible and cost-effective.

Addressing these challenges could make multidisciplinary evaluation and screening a key part of pediatric preventive care, potentially enhancing cardiovascular health in young populations.

## Figures and Tables

**Table 1:** Selection criteria.

**Inclusion Criteria**
Article type: original study, journal article, scientific article Article scope: article reports ECG screening for precocious detection of cardiac pathologies Participants: humans, children (6–12 years), and adolescents (13–18 years) Area of application: articles that conducted research in the field of screening and sports pre-participation evaluation Language: English Publication period: last 20 years
**Exclusion Criteria**
Article type: opinion, case report, commentary, letter to the editor, or conference paper Article scope: not related to ECG Participants: animals, newborns and children <6 years, and adults Area of application: not related to screening cardiac pathologies and sports Language: not in English Publication period: published >20 years ago

**Table 2:** Main measures for children and adolescents (<14 years) and high school students (12–18 years); BMI = body mass index; BP = blood pressure.

n	Measure	Description
A	Gender	To analyze sex-related differences in cardiovascular risk profiles
B	Age	Chronological age documented to stratify findings by developmental stage.
C	Health questionnaire	A structured questionnaire completed by parents or students, assessing personal and family history of cardiovascular symptoms or known conditions.
D	Physical examination (BMI, BP, auscultation)	Body size and standard cardiac examination conducted by trained healthcare professionals, including auscultation and assessment of physical signs.
E	Electrocardiography	12-lead ECG performed to identify electrical abnormalities.
F	Echocardiography	Ultrasound imaging used selectively to evaluate structural heart anomalies.
G	Phonocardiography (PCG)	Recording of heart sounds to detect murmurs and acoustic anomalies.

## Data Availability

The original contributions presented in this study are included in the article. Further inquiries can be directed to the corresponding author.

## Supplementary Materials

The following supporting information can be downloaded at: https://www.mdpi.com/article/10.3390/children12111468/s1, PRISMA 2020 Checklist.

## Abbreviations

The following abbreviations are used in this manuscript:

AHA	American Heart Association
CHD	Congenital Heart Disease
APHRS	Asia Pacific Heart Rhythm Society
CCFT	Cardiac Children’s Foundation Taiwan
CVD	Cardiovascular Disease
ECG	Electrocardiography
ESC	European Society of Cardiology
EHRA	European Heart Rhythm Association
HRS	Heart Rhythm Society
LQT	Long-QT Syndrome
YPPHE	Youth Physical and Public Health Evaluation
PAQ-C	Physical Activity Questionnaire for Older Children
PCG	Phonocardiography
RHD	Rheumatoid Heart Disease
PPCE	Pre-Participation Cardiovascular Evaluation
SCD	Sudden Cardiac Death
TTE	Transthoracic Echocardiography
WPW	Wolff–Parkinson–White Syndrome

## Appendix A

Table A1 reports some specific results of the most notable literature inquiries conducted. The MESH combination “Universal ECG Screening” is more targeted and shows a higher volume of results, with 16 articles and 76 reviews across PubMed and Scopus, including 4 recent publications (2024–2025). This indicates a growing interest in the broader use of ECG, possibly driven by high-profile studies on early detection of inherited cardiac conditions or increased focus on asymptomatic pediatric populations. The large number of reviews (particularly 75 in PubMed) may reflect ongoing assessments of evidence rather than new empirical studies, highlighting the controversial or evolving nature of the subject. The most substantial body of literature is found under the MESH combination “Sports Pre-participation Evaluation,” with 208 (403 PubMed + 5 Scopus) articles and 53 reviews (49 PubMed + 4 Scopus, respectively) and an additional 481 references from Google Scholar (that does not differentiate between articles and reviews). Among these, 66 articles, seven reviews, and five recent studies (2024–2025) were restricted to children and adolescents, indicating this remains an active and expanding research area. This pattern aligns with rising global awareness of SCD in young athletes. The large volume of empirical studies suggests a more established research foundation, with ongoing advances in ECG interpretation criteria, risk stratification, and implementation practices.

**Table A1:** Query results, articles, or reviews from the three search engines. Shaded cells represent the total number of manuscripts in 2024–2025, per database; in round brackets are reported the results only with restriction to “Humans”, “Child: 6–12-year-old”, and “Adolescent: 13–18-year-old”; *, the restriction to the term “Scientific” does not differentiate between articles and reviews; ^, no data from the Google Scholar database are included in the total.

2005–2025	ECG and “Universal Screening”			“Universal ECG Screening”			“Sports Pre-Participation Evaluation”		
	Article	Review	2024–2025	Article	Review	2024–2025	Article	Review	2024–2025
PubMed	9	3	0	12	75	4	203 (66)	49 (7)	19 (5)
Scopus	5	4	0	4	1	0	5	4	0
Google Scholar *	481		77	33		7	481		77
TOTAL ^	14		0	16		4	66		0

## Appendix B. International Societies and Screening Guidelines

AHA: American Heart Association. Refers to the “14-Element Recommendations for Preparticipation Cardiovascular Screening of Competitive Athletes,” designed to identify cardiovascular conditions associated with increased risk of sudden cardiac death in young athletes.

YPPHE: Youth Physical and Public Health Evaluation. A national Italian program aimed at promoting early cardiovascular screening and healthy lifestyles among adolescents through school-based interventions and pre-participation evaluations.

ESC: European Society of Cardiology. Refers to the ESC recommendations and consensus statements on cardiovascular evaluation in young athletes, particularly in the context of sports cardiology and sudden cardiac death prevention.

*ESC Guidelines.*

Screening modalities for cardiovascular disease in young athletes were described in paragraph 3.6 of ESC Guidelines on sports cardiology and exercise in patients with cardiovascular disease [34]; it discusses the importance of early detection of potentially fatal cardiovascular (CV) conditions in athletes and how screening methods compare in effectiveness. (The ESC defines an athlete as “an individual of young or adult age, either amateur or professional, who is engaged in regular exercise training and participates in official sports competition”).

*Early Detection Benefits.*

Most experts agree that detecting CV disorders early can help reduce morbidity (illness) and mortality (death) in athletes. This is achieved through risk stratification (identifying high-risk individuals), disease-specific treatments, and modifications in exercise routines to prevent complications.

*Challenges in CV Screening.*

Screening can be performed using medical history, physical examination, or an electrocardiogram (ECG). Medical history and physical exams often have low sensitivity (they may miss actual conditions). Many pre-participation history questionnaires have a high positive response rate, meaning they frequently suggest potential issues even in healthy individuals, leading to unnecessary follow-ups.

*Effectiveness of ECG Screening.*

Studies show that when trained clinicians use modern ECG interpretation guidelines, ECG screening is more accurate than history and physical exams. ECG has higher sensitivity and better overall statistical performance, meaning it is more effective at detecting heart conditions that could pose a risk to athletes.

## Appendix C. High-Risk of Sudden Cardiac Disease Conditions

A high risk of sudden cardiac diseases was defined by Liu et al. [20] when they found these conditions: (1) structural heart anomalies including dilated cardiomyopathy, hypertrophic cardiomyopathy, restrictive cardiomyopathy, pulmonary hypertension, Eisenmenger’s syndrome, Marfan’s syndrome, and congenital complex heart disease; (2) heart electrical disorders including atrial fibrillation, atrial flutter, complete atrioventricular block, long QT syndrome, ventricular fibrillation, ventricular tachycardia, Wolff–Parkinson–White syndrome, Brugada syndrome, supraventricular tachycardia, and arrhythmogenic right ventricular dysplasia.

## Appendix D. Criteria for Positive History, Physical Examination, and 12-Lead Electrocardiogram at Pre-Participation Screening

These four points were adapted from [34] American Heart Association/European Society of Cardiology guidelines for cardiovascular pre-participation screening in athletes, Italian recommendations for sports eligibility [35], and Bethesda Conference [36,37].

1. *Family History:* (cardiomyopathy, coronary artery disease, Marfan syndrome, long QT syndrome, severe arrhythmias, or other disabling cardiovascular disease);
2. *Personal History:* (i.e., syncope, exertional chest pain or discomfort, shortness of breath, or fatigue out of proportion to the degree of physical effort, palpitations or irregular heartbeat);
3. *Physical Examination:* (i.e., heart murmurs, irregular heart rhythm, brachial blood pressure);
4. *Electrocardiogram:* (atrial enlargement, axis deviation, voltage criteria, conduction abnormalities, repolarization abnormalities, arrhythmias).

Note: These criteria are designed to identify athletes at increased risk for cardiovascular events during sports participation. Positive findings warrant further cardiac evaluation before clearance for competitive athletics [38].
